# Combined Endoscopic Cyclophotocoagulation and Phacoemulsification Versus Phacoemulsification Alone in the Glaucoma Treatment: A Systematic Review and Meta-Analysis

**DOI:** 10.7759/cureus.55853

**Published:** 2024-03-09

**Authors:** Dillan Cunha Amaral, Ricardo Noguera Louzada, Pedro Henrique Santana Moreira, Lucas Neves de Oliveira, Thaís Tiemi Yuati, Jaime Guedes, Milton Ruiz Alves, Denisse Josefina Mora-Paez, Mário Luiz Ribeiro Monteiro

**Affiliations:** 1 Faculty of Medicine, Universidade Federal do Rio de Janeiro, Rio de Janeiro, BRA; 2 Faculty of Medicine, Universidade de São Paulo, São Paulo, BRA; 3 Faculty of Medicine, Faculdade Zarns, Salvador, BRA; 4 Faculty of Medicine, Universidade Estadual de Feira de Santana, Feira de Santana, BRA; 5 Faculty of Medicine, Faculdade de Medicina de Jundiaí, Jundiaí, BRA; 6 Ophthalmology, Glaucoma Research Center, Wills Eye Hospital, Philadelphia, USA

**Keywords:** systematic review, meta-analysis, combined surgery, cataract, glaucoma, phacoemulsification, endoscopic cyclophotocoagulation

## Abstract

The complete safety and efficacy of endoscopic cyclophotocoagulation (ECP) remain unclear in the literature and, to our knowledge, there are no current meta-analyses on phaco-ECP versus phacoemulsification alone to date. Thus, we conducted a systematic review and meta-analysis comparing these two strategies through studies, assessing the effectiveness and safety of outcomes in a population with glaucoma. The protocol for this systematic review was registered in the PROSPERO International Prospective Register of Systematic Reviews (CRD42023482376). We systematically searched PubMed, Embase, and Web of Science from inception to December 2023. A random-effects model was used for all analyses due to heterogeneity. Review Manager 5.3 (Cochrane Centre, The Cochrane Collaboration, Denmark) was used for statistical analysis. Finally, nine studies were included in this comprehensive review and a total of 5389 eyes were analyzed in our study. In comparison to the ECP and phacoemulsification group, those receiving phacoemulsification alone showed better results in best-corrected visual acuity (MD 0.09; CI 95% 0.03 to 0.16; I²=0%), but worse outcomes in intraocular pressure (IOP) (MD -1.49; 95% CI -2.29 to -0.68; I²=29%) and use medications (MD -0.75; 95% CI -0.94 to -0.56; I²=0%) in the last visit. Complication rates, both general and serious, were significantly different between the groups, indicating the potential impact of combined procedures on patient outcomes. Thus, combining ECP with phacoemulsification for glaucoma treatment showed sustained IOP reduction and decreased medication dependence. However, higher complication rates suggest careful consideration of risks. More extensive research with larger trials and longer follow-ups is needed to validate findings and address limitations, providing valuable insights into this treatment approach.

## Introduction and background

Cataract and glaucoma represent two prevalent conditions leading to visual impairment, particularly as individuals age. Given the parallel progression often observed in their natural histories, there is a temptation to address both conditions simultaneously through a single surgical intervention. Cataracts can pose challenges in managing glaucoma by impairing vision, obstructing aqueous outflow, and complicating perimetry and optic disc assessment. Glaucoma encompasses a range of conditions that result in permanent vision impairment, marked by a gradual decline in retinal ganglion cells. While intraocular pressure (IOP) may not always be elevated, extensive clinical trials have identified it as the primary modifiable risk factor. Despite being the foremost cause of irreversible blindness, prompt interventions aimed at reducing IOP have shown efficacy in decelerating the progression of vision deterioration associated with glaucoma [1,2]. Among the various surgical techniques for reducing IOP, the literature highlights phacoemulsification and endoscopic cyclophotocoagulation (ECP) [3-7].

Cataract surgery has demonstrated an additional benefit of reducing IOP in the majority of cases. Increasing evidence supports the use of phacoemulsification either alone or in conjunction with other glaucoma surgical procedures as an effective tool in glaucoma management. Thus, phacoemulsification constitutes a cataract extraction procedure, resulting in a reduction in IOP due to the removal of the lens [8]. Phacoemulsification is generally well tolerated, with postoperative complication rates ranging from 0.1% to 5% in various studies. Achieving a best-corrected visual acuity (BCVA) of 20/60 or better at postoperative week 6 is reported in 98.4% of cases. This favorable risk-benefit profile renders cataract surgery an appealing option for patients with both cataract and glaucoma comorbidities, either as a standalone procedure or in combination with minimally invasive glaucoma interventions [8].

Cyclodestructive procedures were initially recognized for their capacity to lower IOP when Heine observed IOP reductions following detachments of the ciliary body in the early 1900s [9]. In the 1950s, Bietti introduced cyclocryotherapy as a method to reduce IOP. Since then, lasers employing either a transcleral or transpupillary approach have been utilized for IOP reduction. Despite their efficacy in reducing IOP, various cyclodestructive techniques are associated with common complications such as pain, uveitis, prolonged hypotony, hemorrhage, choroidal effusion, and postoperative vision loss [9].

ECP is a more recent FDA-approved technique developed by Martin Uram and offers comparable IOP-lowering benefits without the extensive complication profile associated with other methods. ECP is a procedure that uses laser photocoagulation to ablate the ciliary body processes under direct visualization. This reduces the production of aqueous and helps to lower the IOP. ECP has been proven to be a safe and effective method for reducing IOP. When paired with phacoemulsification, it could be a safer alternative to conventional combined phacotrabeculectomy [10,11]. Moreover, many surgeons have embraced the integration of ECP with phacoemulsification (phaco-ECP) because ECP instrumentation can be seamlessly incorporated into the clear-corneal approach commonly employed in contemporary cataract surgery. Earlier studies have shown that this combined procedure provides a greater decrease in both IOP and the need for glaucoma medications compared to phacoemulsification alone [12]. The emergence of phaco-ECP as a complementary microinvasive technique alongside phacoemulsification is driven by its convenience and minimal risk of complications. Despite its widespread adoption, there is still some variability in the long-term effectiveness data of phaco-ECP in reducing IOP [13].

Thus, the complete safety and efficacy of ECP remain unclear in the literature, and, to our knowledge, there are no current meta-analyses on phaco-ECP versus phacoemulsification alone to date. The rising popularity of combining microinvasive glaucoma surgery with cataract surgery among glaucoma patients has sparked considerable clinical interest in comparing the effectiveness of these combined procedures. Therefore, we conducted a systematic review and meta-analysis comparing phaco-ECP versus phacoemulsification alone, assessing the effectiveness and safety of outcomes in a substantial population.

## Review

Methods

Inclusion and Exclusion Criteria

Inclusion in this meta-analysis was restricted to studies that met all the following eligibility criteria: human study; participants: people with different glaucoma subtypes and cataracts; comparing combined ECP and phacotherapy to phacoemulsification alone; at least one or more interest clinical outcomes; design: randomized trials or nonrandomized cohorts; In addition, studies were included only if they reported any of the clinical outcomes of interest. We excluded studies with no control group; and animal studies or cadaver subjects.

Outcomes

The outcomes of interest were BCVA, IOP, number of medications, and general and serious complications. General complications were defined as secondary glaucoma procedures, cystoid macular edema, corneal edema, fibrin reaction, decentered lens and a capsule, significant inflammation, choroidal detachment, retinal detachment, persistent anterior uveitis, fibrinous uveitis, intracameral tissue plasminogen activator injection, anterior chamber hemorrhage, penetrating keratoplasty, and raised IOP. A subgroup analysis was realized with serious complications defined as cystoid macular edema, fibrin reaction, decentered lens and capsule rupture, significant inflammation, choroidal detachment, retinal detachment, persistent anterior uveitis, fibrinous uveitis, anterior chamber hemorrhage, penetrating keratoplasty, and hemorrhage.

Search Strategy and Data Sources

The protocol for this systematic review was registered in the PROSPERO International Prospective Register of Systematic Reviews (CRD42023482376), and we followed the PRISMA guidelines for protocol data extraction [14]. The term “(Phacoemulsification OR phaco OR "phaco-emulsification") AND ("Cyclophotocoagulation" OR cycloablations OR "Ciliary body ablation" OR “cyclo coagulation” OR "Laser cyclotherapy") AND (glaucoma OR cataract)” was used for the search. We systematically searched PubMed, Embase, and Web of Science from inception to December 2023. The references from all included studies, previous systematic reviews, and meta-analyses were also searched manually for any additional studies. Two authors (D.A. and P.M.) independently extracted the data following predefined search criteria and quality assessment.

Statistical Analysis

This systematic review and meta-analysis were performed in accordance with the Cochrane Collaboration and the Preferred Reporting Items for Systematic Reviews and Meta-Analysis (PRISMA) statement guidelines [15]. Odds ratios (OR) with 95% confidence intervals were used to compare treatment effects for categorical endpoints. Continuous outcomes were compared with mean differences (MD). The forest plots display the MD and standard deviation (SD) for both the "Phaco + ECP" and "Phaco" columns. The measurement of IOP utilized millimeters of mercury (mmHg), while BCVA was assessed using the logMAR scale. The number of injections and complications were recorded as categorical outcomes. Heterogeneity across studies was evaluated using Cochran’s Q test, I2 test, and τ2 test. An I2 value greater than 50% was considered indicative of high statistical heterogeneity, for which a random-effects model was used. A random-effects model was used for all analyses due to heterogeneity [16]. Review Manager 5.3 (Cochrane Centre, The Cochrane Collaboration, Denmark) was used for statistical analysis.

Results

Study Selection and Characteristics

**Figure 1:**
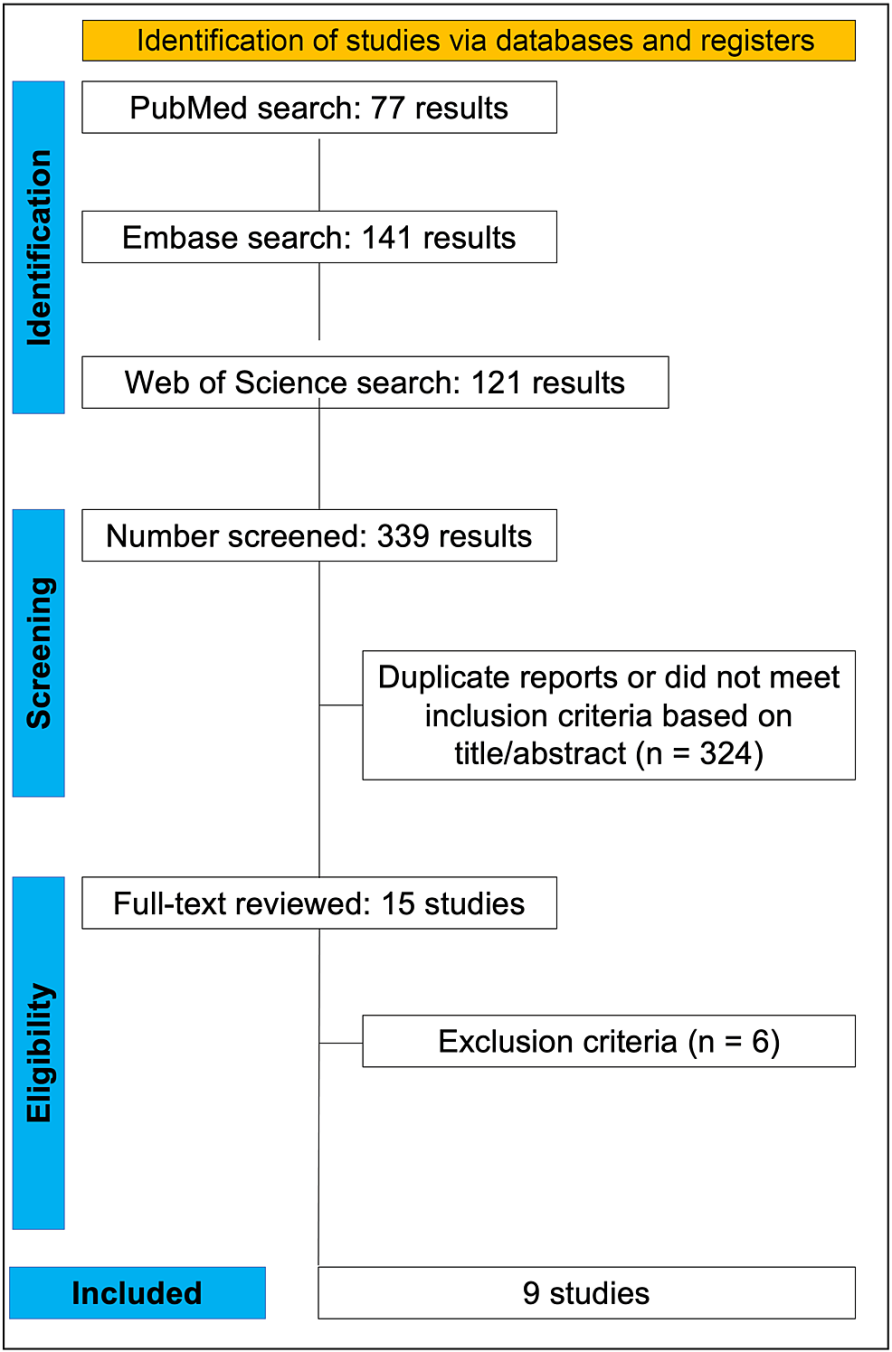
PRISMA flow diagram of study screening and selection PRISMA: Preferred Reporting Items for Systematic Reviews and Meta-Analysis

As detailed in Figure 1, we found 339 articles, with 77 in PubMed, 141 in Embase, and 121 in Web of Science. Of these, 135 were removed as duplicates. After the removal of duplicate records and ineligible studies, 15 remained and were fully reviewed based on inclusion criteria. Next, six articles were excluded as per our exclusion criteria. Finally, nine studies were included in this review, one randomized controlled trial (RCT) [15], one prospective non-randomized cohort [11], and seven retrospective cohorts [16-22].

A total of 5389 eyes were analyzed in our study. The mean age was 73.53 ± 2.60 years in the combined ECP and phaco arm and 72.38 ± 3.63 years in the phaco arm. The number of eyes in the ECP + phaco arm is 806 and, in the phaco, the only arm is 4583. The range of intensity of the ECP laser was 100 - 2000 mW and the range of degree of ECP treatment was 120º - 360º. Study characteristics are reported in Table 1. 

**Table 1:** Baseline characteristics ECP: Endoscopic cyclophotocoagulation; phaco: phacoemulsification; RCT: randomized clinical trial; NA: not applicable.

			Endoscopic cyclophotocoagulation + Phacoemulsification					Phacoemulsification		
Study	Type	Country	Mean age	Eyes	Mean Follow-up	Intensity of the laser	Degree of treatment	Mean age	Eyes	Mean Follow-up
Siegel 2015 [16]	Retrospective	USA	74,8 ± 8	261	36 months	200 - 400 mW	270 - 300	78.1 ± 8.1	52	36 months
Sheybani 2015 [17]	Retrospective	USA	75 ± NA	83	7.4 months	250 mW	243 (120 – 300)	73 ± NA	58	2.3 months
Kang 2017 [18]	Retrospective	UK	76 ± 12	62	21 months	300 - 800 mW	360	74 ± 11	62	21 months
Lai 2021 [15]	RCT	Hong Kong	71.4 ± 9.4	27	24 months	100 - 150 mW	Up to 180	71.2 ± 9.6	21	24 months
Koduri 2021 [19]	Retrospective	USA	71.1 ± 10.9	181	At least 3 months	250 mW	270 - 300	69.9 ± 9.9	4242	At least 3 months
Francis 2014 [11]	Prospective	USA	70.0 ± 6.3	70	36 months	200 mW - 500 mW	270 - 360	69.7 ± 6.9	71	36 months
Bartolomé 2017 [20]	Retrospective	UK	73.94 ± 8.75	69	12 months	250 mW	270 -360	71.6 ± 4.65	30	12 months
Janknecht 2005 [21]	Retrospective	Germany	78.1 ± 7.6	28	5.5 months	1750 mW	NA	77.2 ± 6.8	28	5.5 months
Nirappel 2023 [22]	Retrospective	USA	72.68 ± 9.8	25	8.54 months	140 mW - 400 mW	300 - 360	66.75 ± 9.7	19	3.73 months

Pooled analysis of all studies

Best-Corrected Visual Acuity

**Figure 2:**
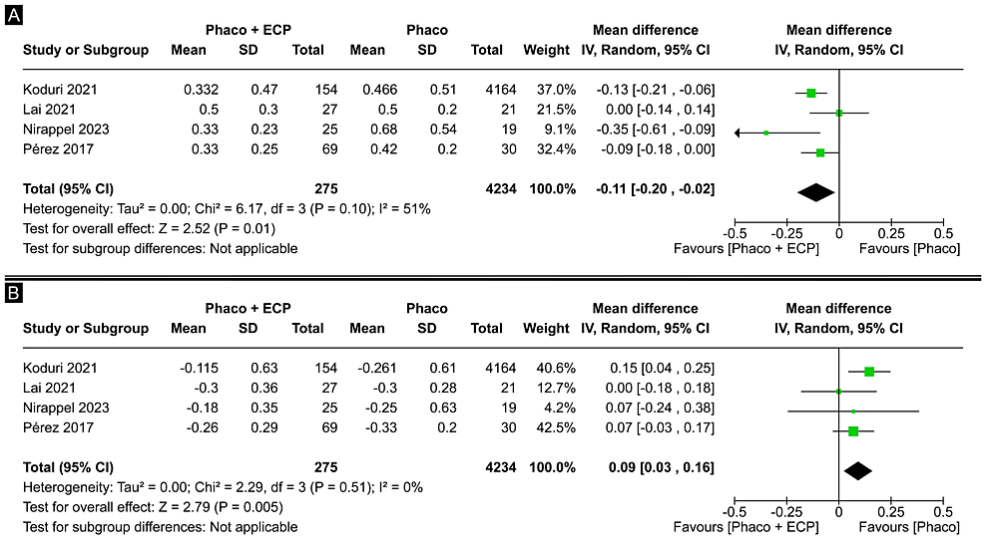
(A) Baseline best-corrected visual acuity forest plot. (B) Mean change best-corrected visual acuity at the final follow-up forest plot. ECP: Endoscopic cyclophotocoagulation

Individuals who received phaco alone had a significant difference in baseline BCVA compared to the group who underwent combined phaco-ECP (MD -0.11 logMAR; CI 95% -0.20 to -0.02; P = 0.01; I²=51%; Figure 2A). In addition, compared to the group who underwent combined phaco-ECP, individuals who received only phaco had a significant difference in the MD in BCVA change at final follow-up (MD 0.09 logMAR; CI 95% 0.03 to 0.16; P = 0.005; I²=0%; Figure 2B).

Intraocular Pressure

**Figure 3:**
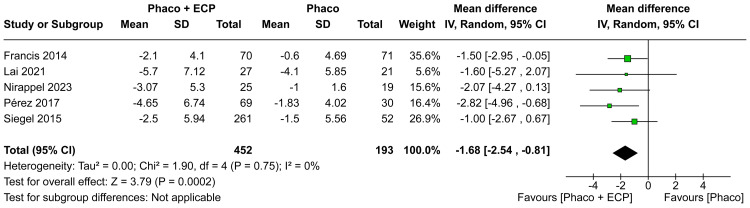
Intraocular pressure in the six-month forest plot ECP: Endoscopic cyclophotocoagulation

**Figure 4:**
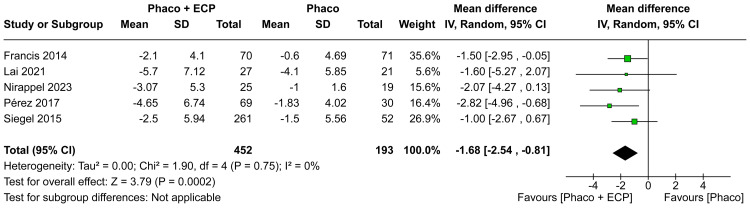
Intraocular pressure in the 12-month forest plot ECP: Endoscopic cyclophotocoagulation

**Figure 5:**
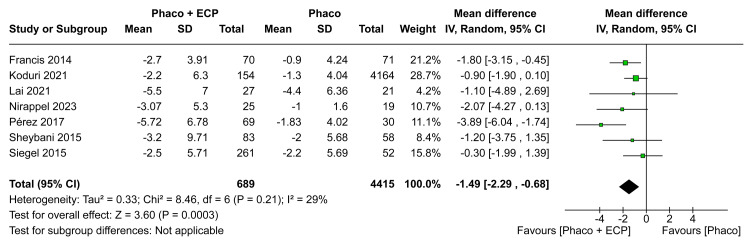
Intraocular pressure in the last visit forest plot ECP: Endoscopic cyclophotocoagulation

In the analysis of the MD in IOP in six months, there was a statistically significant difference between groups (MD -1.84; CI 95% -3.01 to -0.68; P = 0.002; I²=43%; Figure 3). Furthermore, in the analysis of the MD in IOP in 12 months, there was a statistically significant difference between groups between ECP and the phaco group when compared with the phaco (MD -1.68; 95% CI -2.54 to -0.81; p=0.0002; I²=0%; Figure 4). In comparison to the ECP and phacoemulsification group, those receiving phacoemulsification alone had worse results toward an MD of IOP in the last visit (MD -1.49; 95% CI -2.29 to -0.68; p=0.0003; I²=29%; Figure 5). 

Number of Medications

**Figure 6:**
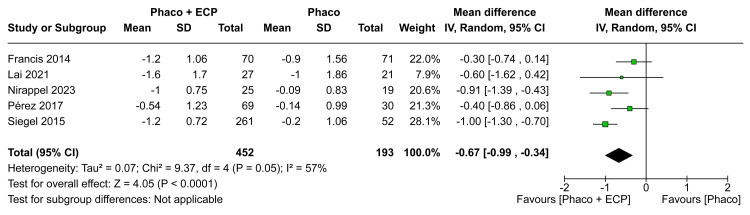
Medications in the six-month forest plot ECP: Endoscopic cyclophotocoagulation

**Figure 7:**
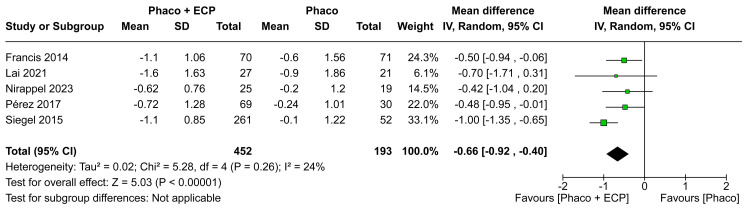
Medications in the 12-month forest plot ECP: Endoscopic cyclophotocoagulation

**Figure 8:**
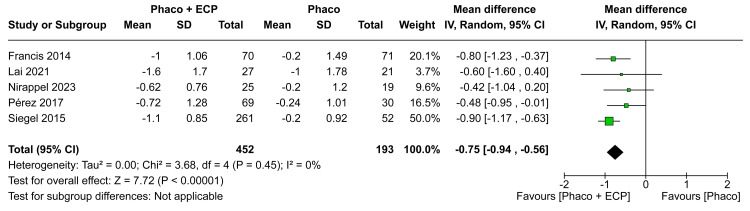
Medications in the last visit forest plot ECP: Endoscopic cyclophotocoagulation

In the analysis of the MD in medications in six months, there was a statistically significant difference between groups (MD -0.67; CI 95% -0.99 to -0.34; P < 0.0001; I²=57%; Figure 6). Furthermore, in the analysis of the MD in medications in 12 months, there was a statistically significant difference between groups between ECP and the phaco group when compared with the phaco (MD -0.66; 95% CI -0.92 to -0.40; p<0.00001; I²=24%; Figure 7). In comparison to the ECP and phacoemulsification group, those receiving phacoemulsification alone had worse results toward a mean difference of medications in the last visit (MD -0.75; 95% CI -0.94 to -0.56; p<0.00001; I²=0%; Figure 8).

Complications

**Figure 9:**
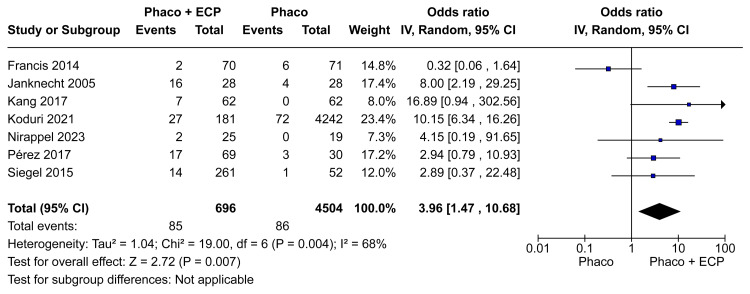
General complications in the last visit forest plot ECP: Endoscopic cyclophotocoagulation

**Figure 10:**
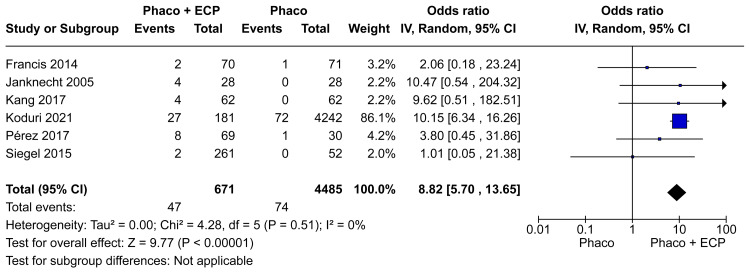
Serious complications in the last visit forest plot ECP: Endoscopic cyclophotocoagulation

Across the pooled studies, a total of 171 complications were observed among the 5200 patients. The phaco-ECP group with 696 patients reported seven secondary glaucoma procedures, nine cystoid macular edema, five corneal edema, seven fibrin reactions, two decentered lens and a capsule, two significant inflammations, one choroidal detachment, three retinal detachments, 37 persistent anterior uveitis, one fibrinous uveitis, one intracameral tissue plasminogen activator injection, four anterior chamber hemorrhage, one penetrating keratoplasty, and five raised IOP. The phaco group with 4504 patients reported five cystoid macular edema, four corneal edema, two significant inflammation, one hemorrhage, 73 persistent anterior uveitis, and one raised IOP. Rates of general complications (OR 3.96; 95% CI 1.47 to 10.68; p=0.007; I²=68%; Figure 9) were significantly different between groups. In the same way, the rates of serious complications (OR 8.82; 95% CI 5.70 to 13.65; p=0.03; I²=0%; Figure 10) were significantly different between groups.

Discussion

This meta-analysis of combining ECP with phacoemulsification versus phacoemulsification alone for glaucoma treatment is the first one in the literature. There is increasing evidence to suggest that phacoemulsification, alone or in combination with other glaucoma surgical procedures, can be a useful tool in treating glaucoma. However, the use of phacoemulsification alone as a standard procedure for glaucoma management is still controversial. In our meta-analysis, the study selection and characteristics are outlined, and 9 studies were included in the review, analyzing a total of 5389 eyes. The mean age, treatment parameters, and serious complications were reported. In comparison to the ECP and phacoemulsification group, those receiving phacoemulsification alone showed better results in BCVA change but worse outcomes in IOP and use of medications at follow-up. Complication rates, both general and serious, were significantly different between the groups, indicating the potential impact of combined procedures on patient outcomes.

Even though baseline BCVA was worse in the phaco alone group (MD -0.11 logMAR; CI 95% -0.20 to -0.02; Figure 2A), the analysis of BCVA revealed that the group undergoing only phacoemulsification exhibited more favorable BCVA change at the final follow-up compared to the ECP and phaco group (MD 0.09 LogMAR; CI 95% 0.03 to 0.16; I²=0%; Figure 2), as suggested by Sheybani et al. [17] This surprising result prompts further exploration into the factors influencing postoperative visual acuity. Sheybani et al. proposed that patients undergoing ECP and phaco may experience a marginally more significant degree of myopia and increased variability in postoperative refraction, potentially attributed to complications specific to the combined procedure [17]. Notably, severe glaucoma patients, often associated with a guarded prognosis, might contribute to this divergence [13,19,21]. Corneal edema induced by intraocular laser in phaco-ECP is a factor that may reduce visual acuity compared to phaco alone, stemming from increased intraoperative corneal distortion and heightened postoperative inflammation [23,24]. The procedure-specific complications in the ECP and phaco group could have played a role in this disparity, underscoring the need for a more comprehensive analysis of the multifaceted factors influencing visual outcomes.

The assessment of IOP consistently demonstrated favorable outcomes across all visits. At six months, the ECP and phaco group exhibited a significant reduction in IOP compared to the phaco-only group (MD -1.84 mm Hg; CI 95% -3.01 to -0.68; I²=43%; Figure 3). This advantage persisted at 12 months (MD -1.68 mm Hg; 95% CI -2.54 to -0.81; I²=0%; Figure 4), indicating the sustained efficacy of combined procedures in lowering IOP. Even in the last visit, the ECP and phaco group maintained favorable results (MD -1.49 mm Hg; 95% CI -2.29 to -0.68; I²=29%; Figure 5). This consistent trend aligns with findings in the literature, suggesting a long-term beneficial effect of the combined approach [13, 21, 25-27]. The modulating properties of ECP on aqueous humor production may elucidate the enduring benefits of IOP reduction over time. This phenomenon becomes particularly noteworthy when compared to outcomes from isolated procedures. The sustained efficacy observed in our study underscores the potential of combined ECP and phacoemulsification in achieving prolonged and significant reductions in intraocular pressure [3].

The analysis of medication management consistently revealed significant benefits over the visits. After six months, we observed a statistically significant reduction in the need for medications in the ECP and phaco group compared to the phaco-only group (MD -0.67 eye drops; CI 95% -0.99 to -0.34; I²=57%; Figure 6). This favorable trend persisted at 12 months (MD -0.66 eye drops; 95% CI -0.92 to -0.40; p<0.00001; I²=24%; Figure 7) and the last visit (MD -0.75 eye drops; 95% CI -0.94 to -0.56; p<0.00001; I²=0%; Figure 8) indicating a continuous advantage of the combination of ECP and phacoemulsification in reducing medication dependence. These results align with existing literature, emphasizing the efficacy of ECP in reducing aqueous humor production and, consequently, decreasing medication use and the future necessity of medications over the long term [3,12,13,17,28-30]. Adjustments in medication management following combined surgeries may be associated with the synergy of ECP's medication-reducing effects and the positive impact on overall treatment effectiveness.

The literature lacks clarity regarding the comparative rates of serious complications associated with phaco-ECP versus phaco-only procedures. In our analysis, we use the definition of serious complications according to Francis et al. [11]. Examining complications across our meta-analysis, encompassing 5200 patients, revealed a total of 171 cases. General complications (OR 3.96; 95% CI 1.47 to 10.68; I²=68%; Figure 9) and serious complications OR 8.82; 95% CI 5.70 to 13.65; I²=0%; Figure 10) were significantly higher in the ECP and phaco group when compared with the phaco-only group. We believed that the observed outcome was probably due to a combination of increased laser energy administered to the eye and the inflammatory damage to ciliary processes, which in turn led to a heightened inflammatory reaction [10,20]. Despite phaco-ECP having a higher number of complications, in general, these were easily treated and nearly all fully resolved with medical and just seven cases required further operative intervention in this meta-analysis [13,19].

Limitations

The study carries some limitations that need to be highlighted for future studies. Our analysis included a relatively small number of RCTs due to the limited research on phacoemulsification combined with ECP. Furthermore, there are a relatively small number of patients in the phaco-ECP group when compared with the phaco group alone. The definition of different types of glaucoma varies greatly among studies, leading to a lack of consensus; despite age and sex matching, the glaucoma severity may be more advanced in one group than another, potentially affecting the baseline IOP values and the number of glaucoma medications. Random-effect models were used in all outcomes to prevent variability in treatment responses across studies. Most studies are retrospective and may have a potential selection bias in severe glaucoma patients who are often associated with a guarded prognosis. Many studies included in this analysis reported follow-up periods ≥ 3 months. However, long-term outcomes (≥ 12 months) beyond the follow-up period were assessed in just a few studies [9,17,19]. It is crucial to assess the long-term stability of surgical effects. Furthermore, there was also no set protocol for discontinuation of preoperative glaucoma medications or initiation of additional glaucoma medications in the postoperative period. The meta-analysis relied on aggregated data from published studies, and individual patient data were unavailable for analysis, limiting a more detailed understanding of the treatment effects and predictors of response.

## Conclusions

In conclusion, the phaco-ECP approach provides an additional opportunity for positive outcomes in terms of sustained IOP reduction and decreased medication dependence when compared with phacoemulsification alone. However, the higher complication rates in the combined group suggest the need for careful consideration of the risk-benefit profile for individual patients. Further research, particularly with larger RCTs and longer-term follow-up, is warranted to validate these findings and address the identified limitations. The results of this meta-analysis contribute valuable insights into the potential advantages and challenges associated with combining ECP with phacoemulsification for glaucoma treatment.
